# Heterogeneity of mammary lesions represent molecular differences

**DOI:** 10.1186/1471-2407-6-275

**Published:** 2006-12-05

**Authors:** Ruria Namba, Jeannie E Maglione, Ryan R Davis, Colin A Baron, Stephenie Liu, Condie E Carmack, Lawrence JT Young, Alexander D Borowsky, Robert D Cardiff, Jeffrey P Gregg

**Affiliations:** 1 Department of Pathology and Laboratory Medicine, School of Medicine, University of California at Davis, Sacramento, CA, 95817, USA; 2 Center for Comparative Medicine, University of California at Davis, Davis, CA 95616, USA; 3 Agilent Technologies, Deer Creek Rd, Palo Alto, CA 94304, USA

## Abstract

**Background:**

Human breast cancer is a heterogeneous disease, histopathologically, molecularly and phenotypically. The molecular basis of this heterogeneity is not well understood. We have used a mouse model of DCIS that consists of unique lines of mammary intraepithelial neoplasia (MIN) outgrowths, the premalignant lesion in the mouse that progress to invasive carcinoma, to understand the molecular changes that are characteristic to certain phenotypes. Each MIN-O line has distinguishable morphologies, metastatic potentials and estrogen dependencies.

**Methods:**

We utilized oligonucleotide expression arrays and high resolution array comparative genomic hybridization (aCGH) to investigate whole genome expression patterns and whole genome aberrations in both the MIN-O and tumor from four different MIN-O lines that each have different phenotypes. From the whole genome analysis at 35 kb resolution, we found that chromosome 1, 2, 10, and 11 were frequently associated with whole chromosome gains in the MIN-Os. In particular, two MIN-O lines had the majority of the chromosome gains. Although we did not find any whole chromosome loss, we identified 3 recurring chromosome losses (2F1-2, 3E4, 17E2) and two chromosome copy number gains on chromosome 11. These interstitial deletions and duplications were verified with a custom made array designed to interrogate the specific regions at approximately 550 bp resolution.

**Results:**

We demonstrated that expression and genomic changes are present in the early premalignant lesions and that these molecular profiles can be correlated to phenotype (metastasis and estrogen responsiveness). We also identified expression changes associated with genomic instability. Progression to invasive carcinoma was associated with few additional changes in gene expression and genomic organization. Therefore, in the MIN-O mice, early premalignant lesions have the major molecular and genetic changes required and these changes have important phenotypic significance. In contrast, the changes that occur in the transition to invasive carcinoma are subtle, with few consistent changes and no association with phenotype.

**Conclusion:**

We propose that the early lesions carry the important genetic changes that reflect the major phenotypic information, while additional genetic changes that accumulate in the invasive carcinoma are less associated with the overall phenotype.

## Background

The paradigm that cancer progression is a multi-step process, associated with multiple molecular changes as it progresses from preneoplasia to invasive carcinoma [1], has been challenged by recent molecular data. Gene expression profiling and comparative genomic hybridization (CGH) studies of breast cancer demonstrate that early stages in the human breast cancer such as ductal carcinoma in situ (DCIS), a precursor lesion for invasive carcinoma, has most, if not all, of the molecular attributes of the corresponding invasive carcinoma despite the distinct pathological characteristics [2-5]. This is contrary to the multi-step paradigm that centers on cumulative molecular aberrations with progression. These data suggest an alternative view that the early lesions are already equipped with the molecular changes responsible for tumorigenesis, despite the disparate histological characteristics between the early lesions and the invasive carcinoma.

Breast cancer can be viewed as a heterogeneous disease histopathologically, as well as molecularly. Molecular profiling studies of breast cancer have shown that tumors can be classified into subtypes based on their expression patterns [6-8]. Pathologically, breast lesions are classified by different categories, such as estrogen receptor (ER) status, Her2 status and the degree of differentiation (tumor grade). Both ER and Her2 status are important prognostic factors and portend how a lesion responds to different therapeutic strategies. Both DCIS and invasive ductal carcinoma (IDC) are categorized into three tumor grades [9]. DCIS lesions are also classified into different subtypes by their histological morphology [10]. It is believed that the different classes of lesions have common characteristics that reflect certain clinical outcomes. Gene expression studies of different pathological stages of breast cancer have shown that different tumor grades are associated with distinct expression signatures, confirming the molecular basis for the differences in pathological classification; the same studies show that the profiles of the different stage lesions also have extensive similarities, suggesting that there may not be as many molecular changes associated with tumor progression as was previously thought [4].

Over the last several years, the MIN-O (mammary intraepithelial neoplasia outgrowth) mouse model has been shown to parallel various aspects of human breast cancer development [11,12]. MIN (mammary intraepithelial neoplasia) is an early mammary lesion that satisfies the operational definition of premalignancy [13]. The MIN-O mouse was established by transplanting a MIN lesion from a young polyomavirus middle-T (*PyVmT*) transgenic female mammary fat pad to a host fat pad [14]. The transplanted MIN lesion will grow from the transplanted location to fill the host fat pad. The growth from the transplanted MIN lesion is called MIN-outgrowth (MIN-O). Since the MIN-O mouse eventually develops invasive carcinoma within the MIN-O, tumor in this context refers to invasive carcinoma that emerges within the MIN-O tissue. Different lines of MIN-O mice, which were established by transplanting MIN lesions to separate hosts, have characteristic histopathology, metastatic potential, and tumor latency, suggesting that MIN lesions have different biological outcomes despite the same initiating oncogene (PyVmT) [11]. In addition, in depth gene expression studies in one MIN-O mouse line, demonstrated that the MIN-O and tumor tissues show strikingly similar expression profiles between each early premalignant lesion and its corresponding tumor pair [12]. Thus, this model recapitulates the foundation of human DCIS and the heterogeneous nature of the disease.

In the current study, we assessed the correlation between certain biological outcomes to molecular changes associated with four different lines of MIN-O mice. In addition to the gene expression analysis, we report for the first time the utilization of high resolution array comparative genomic hybridization (aCGH) to investigate whole genome aberrations in both the MIN-O and tumor tissue. In addition to this whole genome analysis (35 kb resolution), interstitial deletions were verified with a custom made array designed to interrogate the specific regions at approximately 550 bp resolution. We demonstrate that expression and genomic changes are present in the early premalignant lesions and that these molecular profiles can be correlated to phenotype (metastasis and estrogen responsiveness). Progression to invasive carcinoma was associated with few additional changes in gene expression and genomic organization. Therefore, in the MIN-O mice, early premalignant lesions have the major molecular and genetic changes required and these changes have important phenotypic significance. In contrast, the changes that occur in the transition to invasive carcinoma are subtle, with few consistent changes and no association with phenotype. Therefore, we propose that the early lesions carry the important genetic changes that reflect the major phenotypic information, while the additional genetic changes accumulated in the invasive carcinoma are less associated with the overall phenotype.

## Methods

### Mice

Standard techniques for mammary gland clearing and mammary tissue transplantation, and the establishment of the MIN-O lines, have been described previously [11,12]. Three-week-old FVB female mice were purchased from Charles River Laboratories (Wilmington, MA). Animals were maintained in a vivarium at the University of California Davis campus under IACUC approved protocols.

### Ovariectomy

To test MIN-O ovarian hormone sensitivity, a 1 mm^3 ^piece of a MIN-O tissue from each line was transplanted in the gland-cleared #4 fat pads of 3-week-old virgin FVB female mice. At the time of transplantation, the experimental group (ovx, n = 8) was ovariectomized, while the control group (intact, n = 4) received mock surgery without ovariectomy. At 5 weeks after transplantation, the MIN-O sizes were measured by visually inspecting the transplantation site under a dissecting microscope. Animals were palpated weekly until a tumor was detected. The experiment was concluded at day 99 post-transplantation. For the tumor ovarian hormone sensitivity experiment, 1 mm^3 ^pieces of tumor tissue that developed from various MIN-O lines were transplanted to intact and ovx hosts (at least 2 animals/4 tumors each for a tumor). Both intact and ovx animals were terminated at the same time and the tumor bearing fat pads were weighed.

Immunohistochemistry of formalin fixed tissues using anti-estrogen receptor was performed as previously described [15]. MIN-O transplanted fat pads from the ovariectomy experiment were used to determine the ER localization in the MIN-Os from the intact and ovariectomized hosts.

### Mouse Genome CGH Microarrays

The Agilent Mouse Whole Genome CGH Microarray Kit 44A (G4414A, Agilent Technologies, Santa Clara, CA) consists of roughly 43,000 60-mer oligonucleotide probes that cover both exonic and intronic sequences. These arrays were used to analyze the MIN-Os and tumors from each line. These high resolution arrays have an average spatial resolution of 35 kB, after removal of Repeat Masked (-RM) [16]. Agilent uses SurePrint^® ^technology [17] to print this entire content on a standard 1" × 3" glass slide.

In order to better define the length and boundaries of interstitial regions that were identified as deleted or amplified with the CGH 44A Microarray, a custom array was generated to specifically interrogate these regions at a higher resolution. Four regions were selected for analysis (see Table 1). Approximately 38 K probes were used to cover these regions for an average spacing of 547 bp (-RM). Chromosomes 7 and 8 did not show any interstitial aberrations or whole chromosome aberrations, so 4990 probes on these two chromosomes were utilized for the normalization. In total, each "zoom-in" array contained 44,000 unique probes.

DNA was extracted from frozen MIN-O/tumor pairs isolated from the same fat pads of MIN-O lines 4w-4 (n = 5 pairs), 4w-11 (n = 3 pairs), 8w-B (n = 3 pairs) and 8w-D (n = 3 pairs). DNA from FVB/NCrl female livers was used as the reference sample. Samples were prepared using the direct labeling method, according to the Oligonucleotide Array-Based CGH for Genomic DNA Analysis Protocol (Version 2.0, available at [18]), with minor modifications of the protocol. A total of 2 ug of digested purified DNA was labeled, using the BioPrime Array CGH Genomic Labeling kit (Invitrogen, Carlsbad, CA), for both the experimental and reference channel. During the labeling, cyanine 3-dUTP and cyanine 5-dUTP (Perkin Elmer, Wellesley, MA) were incorporated in either the experimental or the reference sample. For each sample, two arrays were utilized in a dye-swap pair format, in which the experimental sample and reference sample are each labeled separately with cyanine 3 and cyanine 5, respectively, and combined for the first array, and then labeled in the reverse and combined for the second array. After labeling, the corresponding experimental and reference samples were combined and unincorporated nucleotides were removed on a Microcon YM-30 apparatus (Millipore, Billerica, MA). The purified sample was then hybridized onto a single CGH array in a solution containing Mouse Cot-1 DNA (Invitrogen, Carlsbad, CA), Agilent Blocking Agent, and Agilent Hybridization Buffer (Agilent, 5188–5226). Hybridization took place in a rotisserie oven set to 65°C with a rotation speed of 20 rpm. The washing and scanning of the slides was performed in an ozone-free area to prevent the degradation of the cyanine 5 dye. Each slide was washed according to the manufacturer's recommendations with no Stabilization and Drying Solution. The slides were scanned on an Agilent DNA Microarray Scanner B.

TIFF image files generated from the scanner were analyzed using Agilent's Feature Extraction Software 8.1. This software allows a standardized method of extracting data using a standard CGH normalization protocol based on all array features (44k_CGH_0605). The QC report generated was checked to ensure proper hybridization and placement of the grid by the software. Data generated by the Feature Extraction tool were loaded into CGH Analytics 3.1 (Agilent Technologies) to allow visualization of the data. Dye swap pairs were combined to facilitate the visualization of chromosomal aberrations. A smoothing average of 50 MB was used for the full genome arrays. Regions of aberration (deleted or amplified) were determined by the methodology of Barrett et al.[19].

### Gene expression

Total RNA extraction and biotin-labeled probe synthesis for gene expression analysis has been described previously [11,12]. Frozen tissues from MIN-O lines 4w-4 (n = 3 MIN-Os, n = 3 tumors), 4w-11 (n = 3 MIN-Os, n = 3 tumors), 8w-B (n = 4 MIN-Os, n = 4 tumors) and 8w-D (n = 4 MIN-Os, n = 3 tumors) and intact mammary fat pads from 16-day prelactating FVB females (n = 2) were used for hybridization onto Murine Genome U74Av2 GeneChips (Affymetrix, Santa Clara, CA). The scanned images were processed using GCOS 1.4, and then the data was analyzed with GC-RMA in the ArrayAssist software package (Stratagene). GCRMA derived expression values were log-transformed and analyzed in Arrayassist (t-test, unequal variance). Expression comparisons of all MIN-Os to prelactating mammary glands, and all tumors to MIN-Os were carried out in Arrayassist using t-test with FDR correction. 938 probes were differentially expressed with p < 0.01 in the MIN-Os vs prelactating comparison. 55 probes were differentially expressed with p < 0.01 in the tumors vs MIN-Os comparison. For expression comparisons of samples from individual lines, profiles were compared in Arrayassist using t-test without FDR correction and gene lists were generated with criteria of p < 0.01.

Gene lists were subjected to pathway and function analysis in Ingenuity Pathways Analysis (Ingenuity Systems, Redwood City, CA). Annotation and gene ontology of each probe was assigned by NetAffyx (Affymetrix, Inc). The results from the statistical analyses described above were analyzed by ErmineJ software [20] to determine the number of significant gene ontology (GO) categories using gene score resampling (GSR) [21]. Unfiltered t-test results were imported into Erminej and analyzed using a 100,00 fold iteration re-sampling approach to determine which Biological Processes categories were significant below a Benjamini-Hochberg FDR-corrected p-value of 0.01.

For correlating the genomic changes to the expression differences, chromosome locations of probes and known genes were determined by the UCSC Genome Browser [22]. For the analysis of whole chromosome gain of chromosome 2, expression values of chromosome 2 probes (n = 871) from 8w-B and 4w-4 MIN-Os were compared to those from 8w-D and 4w-11 MIN-Os (t-test, unequal variance). For chromosome 1, 10 and 11 probes (n = 652, 481, and 957, respectively), expression values from 8w-B MIN-Os were compared to those from 8w-D, 4w-4, and 4w-11 (t-test, unequal variance). For the analysis of 2F, 3E, 17E and 11B, the corresponding probes were identified in UCSC Genome Browser. For 2F, 3E and 17E probes, the average expression values of the 8w-D MIN-Os were compared to the average expression values of 8w-B, 4w-4 and 4w-11 MIN-Os. For 11B probes, expression values of the 5189 MIN-O sample were compared to those of the 5207 MIN-O sample.

## Results

### MINs are heterogeneous

Each transplantable MIN-O mouse line was established from an individual MIN lesion from a Tg(*PyVmT*) mammary fat pad. Each line has characteristic phenotypes (Table 2), as previously described [11]. Distinct histological features have been maintained in each MIN-O line over multiple transplant generations. Detailed histological analysis of MIN-O and tumor tissues from each MIN-O line has been published previously [11]. The metastatic potential of each MIN-O line is also different: 8w-D and 4w-11 MIN-Os are considered metastatic lines, while 8w-B and 4w-4 are non-metastatic lines.

#### Ovarian hormone sensitivity of MIN-Os

ER status and ovarian hormone dependence is an important diagnostic factor for mammary lesions. In general, early mammary lesions developed in Tg(*PyVmT*) mammary fat pads are ER-positive, but the expression of ER is lost as the lesions progress to invasive carcinoma [23]. Previously, we showed that the MIN-O from the 8w-B mouse line was ER-positive and also that MIN-O growth was ovarian hormone-dependent, while the tumors derived from the 8w-B MIN-O express significantly less ER [15]. Since each MIN-O line represents individual mammary lesions from Tg(*PyVmT*) fat pads that have distinct characteristics, we tested the ovarian hormone dependence of the MIN-O lines by transplanting each to the fat pads of ovariectomized host females (Table 3). Ovarian ablation decreased the average MIN-O size and delayed palpable tumor development, but with various degrees of statistical significance in each line. In particular, ovariectomy significantly inhibited both the MIN-O growth and tumor latency in the 8w-B and 8w-D mice, while only the MIN-O growth was significantly affected in the 4w-4 mice. In the 4w-11 mice, the ovariectomy reduced the averaged MIN-O size and increased tumor latency, but the effects were not statistically significant. On the other hand, when tumors that developed in ovary-intact MIN-O mice were transplanted to ovariectomized hosts, the ovariectomy did not inhibit tumor growth significantly in any of the lines (data not shown). This demonstrates that the growth of the tumors that have developed from the MIN-Os is not dependent on ovarian hormones while the growth of the MIN-Os is sensitive to the levels of these hormones.

ER expression in the 4w-4, 4w-11, 8w-B and 8w-D MIN-Os were determined by immunohistochemistry of the MIN-O transplanted fat pads (Table 2). In the intact hosts, patches of strong nuclear ER-positive cells were present in the 8w-B MIN-Os, while in the 8w-D MIN-Os, nuclear ER-positive cells were also present, but with less intensity. In 4w-4 and 4w-11 MIN-Os from the intact hosts, nuclear ER staining was rarely found and, if any, weak ER staining was seen in the cytoplasm. In all four lines, MIN-Os from the ovariectomized hosts had no nuclear ER-positive cells, and reduced cytoplasmic staining, if any, compared to those from the intact hosts (data not shown).

#### CGH analysis

To study genomic aberrations associated with MIN-O and tumor samples, we utilized Agilent mouse CGH arrays on 14 MIN-O/tumor pairs. Genomic DNA from at least 3 different MIN-Os and the corresponding tumors from the same fat pad were isolated from 4 different MIN-O mouse lines.

**Figure 1:**
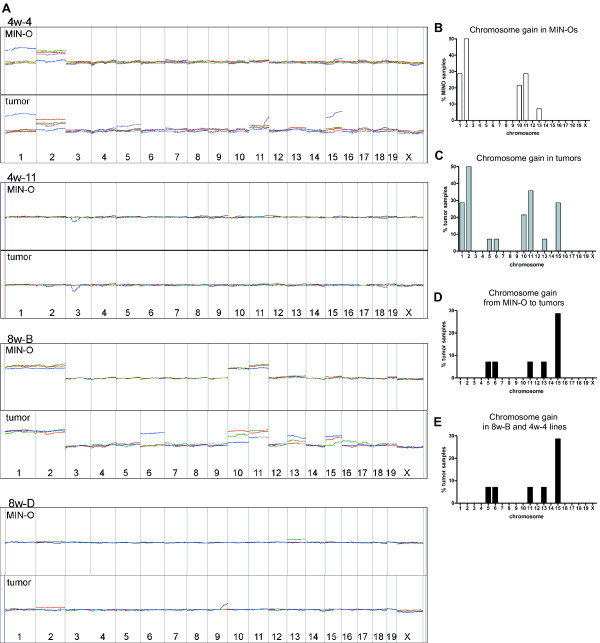
**Mouse whole genome CGH microarray data from MIN-Os and tumors**. A) Summary of array CGH data by MIN-O lines. Agilent mouse whole genome CGH microarray was used for genomic DNA from different MIN-O and tumor pairs from 4w-4, 4w-11, 8w-B and 8w-D MIN-O mice. The graphs were generated with a smoothing average of 50 MB. Each Pair of MIN-O and corresponding tumor from the same animal is represented with the same color (n = 4 for 4w-4, n = 3 each for 4w-11, 8w-B and 8w-D). 4w-4 and 8w-B samples have multiple whole chromosome gains. 4w-11 and 8w-D samples are relatively genetically stable. B-E) Frequency of whole chromosome gains found in MIN-Os and tumors by array CGH. B) Distribution of whole chromosome gains in all MIN-Os studied with array CGH (n = 14). The most frequent gain is found on chromosome 2, followed by chromosome 1 and 11. C) Distribution of chromosome gains in all tumor samples studied with array CGH (n = 14). Tumors have the same whole chromosome gains found in MIN-Os with a few additional gains. D) Frequency of whole chromosome gain events associated with MIN-O to tumor transition. Whole chromosome gains in tumors that are not found in the corresponding MIN-O samples are presumed to be acquired during the transition from MIN-O to tumor. E) Frequency of chromosome gains in 8w-B and 4w-4 samples (n = 8 for MIN-Os and tumors each). The majority of whole chromosome gains were found in the 8w-B and 4w-4 MIN-O lines.

Nine out of 14 MIN-Os carried some type of whole chromosome gain (Figure 1B). The most common chromosome aberration was whole chromosome gain of chromosome 2 (50%), followed by chromosome 11 (28.6%), 1 (28.6%), and 10 (21.4%). The corresponding tumor samples carried the same chromosome gain(s), except for one pair. Some of the tumors had acquired additional genomic changes, suggesting that these changes occurred during the MIN-O to tumor transition (Figure 1C). The most common gain associated with the tumor transformation was the whole chromosome gain of chromosome 15 (4 out of 14 tumors) (Figure 1D). Other whole chromosome gains associated with the progression to tumor were found for chromosome 5, 6, 11, and 13 (1 out of 14 tumors, each). 36% (5/14) of the MIN-Os had no major genomic abnormalities.

In addition to the whole chromosome gains, the MIN-Os also exhibited chromosomal losses and/or gains of small chromosomal regions. The most common regions deleted were 2F1-2 (21.4% MIN-Os and tumors), 3E4 (7.1% MIN-Os, 21.4% tumors), and 17E2 (7.1% MIN-Os, 21.4% tumors). Other regions that were found to have deletions in one MIN-O/tumor pair were 12A, 16C2, and 3D-E1. Also, the deletion of a small region in 19D was found in a single tumor.

Small interstitial gains (duplication) of a chromosome material were not as common as interstitial chromosome deletions. The only recurring small gain was 11B1.2 (7.1% MIN-Os, 14.2% tumors). Another area of interest was duplication of 11E1 found in one tumor sample. This area has been reported to be amplified in Tg(*PyVmT*) tumors [24].

#### Expression changes associated with tumorigenesis

To understand the expression changes associated with mammary tumorigenesis, as well as the molecular basis of the heterogeneity of mammary lesions, we studied gene expression profiles of MIN-Os and tumor samples. Highly proliferative wild-type mammary gland (prelactating) was used as the control, as it has the same proliferative index as the early preneoplastic tissue (in contrast to the non-proliferative quiescent mammary fat pad). The tissues were analyzed with the Affymetrix GeneChip MG-U74Av2 oligonucleotide arrays.

The gene expression profiles of the MIN-Os were dramatically different from that of the proliferating mammary glands. 937 probes (899 genes – comprised of 19 unknown probes, 103 unknown genes or ESTs, and 777 known genes) were differentially expressed (p < 0.01). 293 out of the 777 known genes were down-regulated in MIN-Os and 484 out of the 777 genes were over-expressed in MIN-Os. The majority of these genes were involved in cell cycle and/or cell death functions [see Additional file 1].

In general, the expression profiles of tumors were very similar to those of the MIN-Os. Only 55 probes (50 known genes) were differentially expressed (p < 0.01), and they were all down-regulated (Table 4). Nine of these genes are related to extracellular matrix (TNXB, SGCE, DPT, LUM, GSN, LAMA2, C1R, CLEC3B, DF, LPL, NID, RBP4, RETN); another set of noticeable genes are Igfbp-6 and PPP2R5a, which are anti-proliferative genes. Since many of these genes are known to be found in stroma, we examined the expression level of an epithelial marker, Krt2-8 (Keratin 8), in the samples to determine whether the expression changes are due to the change in the epithelial component of the tissues. Krt2-8 expression between the MIN-Os and tumors was not statistically significantly different (p = 0.1855, t-test, FC = 1.2). In previous studies, TaqMan quantitative PCR was utilized to confirm the expression of several genes (decorin, IGFBP2, Thrsp, Sncg, SerpinG1) in MIN-O and tumor pairs and showed that this data correlated with the direction and fold change of the microarray data [12]. In addition, immunohistochemistry studies demonstrated that the gene expression differences are not due to differences in the epithelial and stromal abundance in the MIN-O and tumor but rather due to inherent differences in the tissues. Thus, it is unlikely that the significant down-regulation of these 50 genes in the tumors is solely due to the enrichment of the stroma in the MIN-Os.

### Molecular changes associated with each MIN-O line

#### Recurring genomic aberration by MIN-O lines

When CGH results were analyzed for each MIN-O line, we found recurring genomic changes that were specific to certain lines. MIN-Os from 8w-B and 4w-4 mice carried large chromosome aberrations, frequently including multiple chromosome gains, while large chromosome aberrations were rare in the 4w-11 and 8w-D lines (Figure 1A&E). 100% of 8w-B MIN-Os carried whole chromosome gain of chromosomes 1, 2, 10 and 11. 80% of 4w-4 MIN-Os carried whole chromosome gain of chromosome 2. Tumors arising from both MIN-O lines (8w-B and 4w-4) often acquired additional chromosome gain(s) (6 out of 8 tumors), although the majority of the cases were limited to a gain of one chromosome, and there were no recurring chromosome gains found in tumors, except for chromosome 15.

In the MIN-O lines with whole chromosome gains, the gene expression pattern reflected the genomic changes. When expression of genes on chromosome 2 from the MIN-O lines with whole chromosome gains (8w-B and 4w-4) were compared to those from the lines with normal chromosome number (4w-11 and 8w-D), 60 probes had significantly higher expression (p < 0.01) in 8w-B and 4w-4 MIN-Os, while only 5 probes had significantly lower expression, when compared to 4w-11 and 8w-D MIN-Os (Table 5). For chromosomes 1, 10 and 11, which were gained in 8w-B MIN-Os, genes on these chromosomes had higher expression in 8w-B MIN-Os compared to the rest of the MIN-Os (Table 6).

In-depth analysis of the data found that all the 8w-D samples carried deletions of 2F1-2, 3E4, and 17E2 regions. A pair of MIN-O and tumor samples from one 8w-D animal (5189) had the same amplification of 11B1 region. We also found a large amplification of 11E in a tumor from one 4w-4 animal. Among the four MIN-O lines, we found the least amount of chromosome abnormality in 4w-11 samples. 4w-11 samples did not carry any whole chromosome aberrations, or large duplications. In one MIN-O/tumor pair of 4w-11, we found a small deletion in the 3D-E1 region.

#### Zoom-in arrays

**Figure 2:**
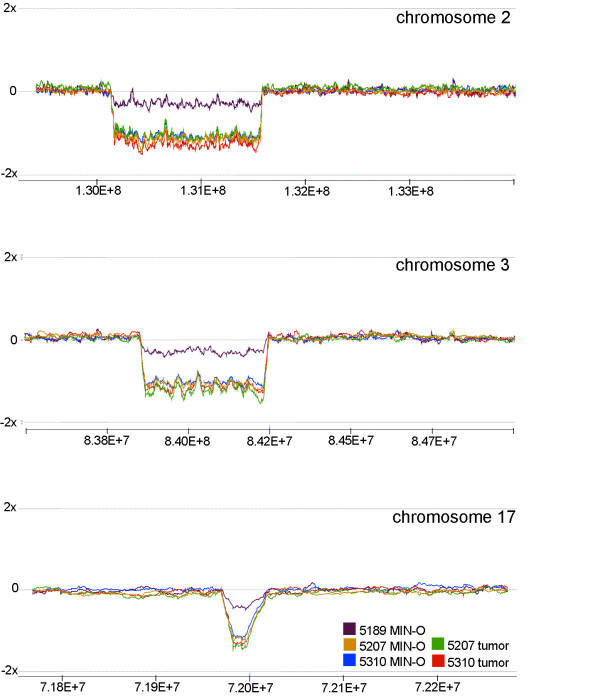
**High resolution custom CGH microarray data on selected regions on chromosomes 2, 3 and 17**. Small deletions in 2F, 3E, and 17E in MIN-Os and tumors from the 8w-D line were confirmed with the custom designed "zoom-in" array with average spacing of 547 bp. The specific breakpoints were determined as follows: chr2:130,621,986-131,735,939, chr3:83,909,586-84,292,657, chr17:72,077,340-72,137,044.

To confirm the small deletions and duplications detected by the whole genome arrays, a custom CGH array for the 11B, 11E, 2F, 3E, and 17E regions was designed (see materials and methods). With this "zoom-in" array, we were able to more finely map the deleted regions in the 8w-D samples as follows: 111kb on 2F1-2 (chr2: 130,621,986-131,735,939), 383 kb on 3E4 (chr3: 83,909,586-84,292,657), 59 kb on 17E2 (chr17: 72,077,340-72,137,044) (Figure 2). The genes in these regions are: Hspa12b, 1700037H04Rik, Spef1, Cenpb, cdc25b, 2310035K24Rik, D430028G21Rik, Pank2, Rnf24, Smox, Adra1d, 1600014E20Rik, Prnp, Rassf2, Slc23a2, on chromosome 2; Trim2, 6330505N24Rik, on chromosome 3; and 2810410M20Rik on chromosome 17. 10 out of 16 genes in the deleted region on chromosome 2 are represented on the 74Av2 array by 10 probes (Table 7). In 6 out of the 10 probes, the average expression in the 8w-D MIN-Os was lower as compared to the MIN-Os from other lines (Table 7). The deleted regions in 3E and 17E were covered by only one probe each (104207_at for 6330505N24 and 98886_at for 2810410M20Rik). The expression of both genes was lower in 8w-D MIN-Os compared to the other MIN-Os.

The detailed breakpoints of the two duplicated regions (11B and 11E) were confirmed by the zoom-in array. The breakpoints on 11qB were 42,631,278-44,744,905, which spanned the following genes: Pttg1, D11Ertd730e, C1qtnf2, Fabp6, Ttc1, Adra1b, Il12b, Ublcp1, 3732413I11Rik, Ebf1 (Table 8). These genes are represented by 11 probes on the 74Av2 array, and the expression of these genes in the 5189 MIN-O (the 8w-D MIN-O sample with the 11B amplification) are higher than in the 5207 MIN-O (an 8w-D MIN-O without the amplification). The 11qE1 region seems to be comprised of regions with varying degrees of amplification, starting from 102,146,284 to the end (121,647,795) (data not shown). In general, the interstitial aberrations identified show concomitant gene expression changes and suggest that there is a DNA dosage-gene expression relationship.

#### Gene expression associated with each MIN-O line

Since each MIN-O line has distinct biological characteristics, we expected to find unique gene expression features for each line. To identify unique expression changes associated with each MIN-O lines, we subjected each list of MIN-O vs. PL differences to GSR GO analysis. There were only 5 significant GO biological function terms (p < 0.05) in the 4w-4 line (DNA-dependent DNA replication, chromatin assembly or disassembly, nucleosome assembly, regulation of cyclin dependent protein kinase activity, organic acid biosynthesis), of which the first four functions were significant in the other lines (Table 9). The 4w-11 line was characterized by three highly significant I-kappaB/NF-kappaB pathway-related functions (p < 0.0001) (Table 10). The cell cycle and mitotic spindle/microtubule related functions were particularly highly represented in 8w-B line (Table 11). The 8w-D line had over-representation of specific signaling pathways (Wnt, Ras and MAPK), as well as ECM-related functions (Table 12). Moreover, certain functions were common in multiple MIN-O lines. The immune response-related functions, as well as the I-kappaB/NF-kappaB cascade related functions, were very highly represented in the 4w-11 and 8w-D lines. The functions related to UV light/radiation response were highly significant in the 4w-11 and 8w-D lines. 8w-B and 8w-D lines shared angiogenesis and steroid biosynthesis related functions as highly significant biological functions

Although the comparison of all tumors vs. all MIN-Os identified a small number of down-regulated genes, there was no one gene common to the tumor vs. MIN-O gene lists from the four MIN-O lines [see Additional file 2]. Because the expression profiles of tumors were very similar to the corresponding MIN-Os, GO analysis did not result in any significant functional terms. However each line had characteristic expression differences of genes that suggest changes in tumor-associated functions. Tumors developed from 4w-4 MIN-Os were characterized by down-regulation of immunoglobulin and up-regulation of actin and myosin (3- to 4-fold change). Tnfrsf11/RANK, receptor activator of NfκB [25], and RalA, which is a critical component for Ras-induced tumorigenesis [26] were also up-regulated in these tumors. In 4w-11 tumors, CEBPA, which is associated with breast cancer progression [27] was down-regulated and CEBPB, a gene found to be up-regulated in breast cancer as well as mouse mammary tumors [28], is up-regulated. Moreover, the 4w-11 tumors exhibited down-regulation of actin, myosin, Ig, and MHC class 2 transcripts, which have been shown to be decreased in highly metastatic breast cancer cell lines [29], as well as decreased during progression to tumor [30]. 8w-B tumors had up-regulation of PLK [31] and 8w-D tumors had up-regulation of Epha2 [32].

### Molecular changes associated with biological characteristics

#### Molecular changes in MIN-Os with genetic instability (lines 8w-B and 4w-4)

dysregulation of mitotic spindle and chromosome segregation related genes To further understand the basis of chromosome instability in the MIN-Os, we assessed gene expression profiles of the genetically unstable MIN-O lines – 8w-B and 4w-4. Seven out of eight MIN-O samples from these two lines had whole chromosome gain of chromosome 2. The two lines had 13 genes from chromosome 2 in common in the gene expression comparison between MIN-O and PL [see Additional file 3]. Among these 13 genes, all but one was over-expressed in the MIN-Os. Aurora kinase A (Aurka/STK6/STK15) was the most dramatically over-expressed (9- to 10-fold).

Aurka regulates centrosome duplication and mitotic spindle assembly. Other genes involved in spindle assembly (Ran, Tuba1, Tubb6, Kif22, Nde1, Ndrg1) and centrosome duplication and sister chromatid segregation (Plk1, Gadd45a, Apc11, Cdc20 and Cdkn1a) were also dysregulated. In addition, genes up-regulated in 8w-B and 4w-4 MIN-Os included a large group of E2F4 regulated genes (H2AFZ, RFC2, RFC4, ZNF305, KIF22, RPA3, RRM1, CDK4, CDC2, PLK1, PSMA4, and TK1) [33].

#### Expression profiles associated with metastatic MIN-Os

8w-D and 4w-11 are the two MIN-O lines with highest metastatic index (Maglione 2004). From our GO analysis (Table 7), MIN-Os from both lines seem to have dysregulation of significant number of genes that are involved in immune-related responses and apoptotic program. The MIN-Os from these two lines share 209 probes that are dysregulated [see Additional file 4]. Among the 209 probes (186 genes and 18 ESTs), 42 genes are unique to these two lines. Examples of these unique genes include Serpine2, which is overexpressed in metastatic cancer; Ctnnb1, Fliih, Pdlim4/Ril, all of which affect migratory behavior of cells; and Gas6, which is a ligand for the Axl oncogene [34].

The metastasis signature genes in human cancer [35] have been reported to be predictive of a high metastatic rate in mouse tumors [36]. 14 out of these 17 signature genes are represented in the MG-U74Av2 array. We compared the expression of these 14 genes in both MIN-Os and tumors, between the two most metastatic MIN-O lines (8w-D and 4w-11) and the two least metastatic MIN-O lines (8w-B and 4w-4). We did not find significant correlation of the expression trends between what has reported with human metastatic breast cancer and our metastatic MIN-O lines (data not shown), yet, further analysis and comparison to the human data is required in order to identify potential correlated GO terms and pathway hubs that may better reflect similarity in the model and the human data.

#### Expression profiles associated with estrogen sensitivity

8w-B and 8w-D MIN-Os are more sensitive to ovarian ablation than other lines. Transcript levels of ERs in the 8w-B and 8w-D MIN-Os tend to be slightly higher than the 4w-4 and 4w-11 MIN-Os [see Additional file 5], which is consistent with ER expression found in the immunohistochemical analysis. Moreover, the expression of cyp19a1, an aromatase which catalyzes essential reactions for estrogen biosynthesis, is also slightly but statistically significantly higher in the 8w-B and 8w-D lines (fold change = 1.07, p = 0.0068). In these lines, steroid biosynthesis (p = 0.0345 for 8w-B, p = 3 × 10^-6 ^for 8w-D) and sterol metabolism (p = 0.0416 for 8w-D) were represented as highly significant GO functions (Table 7). In addition, angiogenesis related functions were also significant. 271 probes (114 probes unique to only these two lines) were differentially expressed in both 8w-B and 8w-D MIN-Os [see Additional file 6]. Genes dysregulated only in 8w-B and 8w-D MIN-Os include proteins that interact with estrogen or ER, such as Abcg1, Gnai2, Fbln1, Nr4a1, Jun (all up-regulated), and Strn and Cyp1b1 (both down-regulated). Also, NfκB pathway genes – Nfkb2, Relb, and Bcl10 – were down-regulated.

## Discussion

### Major chromosome abnormalities associated with tumorigenesis are present at the MIN-O stage

This study is the first molecular analysis of mammary tumor progression in the four MIN-O mouse lines, and it shows that MIN-Os, mouse mammary premalignant lesions, are already genetically and molecularly advanced, with molecular and genomic profiles similar to those of invasive tumors. This study also confirms that these lesions, which originated in the same organ site, are genetically heterogeneous, as reflected from their morphological, genomic, and gene expression characteristics. We hypothesize that even with the same initiating oncogene, the resulting early premalignant lesions and subsequent tumors will have different groups of biologically distinct characteristics that are determined by the genetic changes already present in the early lesions – in this case, in the MIN-Os.

From the whole genome analysis of MIN-O and tumor samples by the array CGH, we found that most lesions already have chromosome aberrations at the MIN-O stage. The results from the expression arrays mirror the CGH results in that major expression changes were seen in the MIN-O stage and expression changes between the MIN-Os and tumors are surprisingly relatively few. The finding that the MIN-Os already have the major genomic and expression changes that have previously been associated with tumorigenesis is consistent with observations in human breast cancer [3,4]. Human DCIS has been found to have the same chromosome alterations found in invasive ductal carcinoma [3]. In addition, at the gene expression level, ADH, DCIS, and IDC from the same individual are highly similar to each other [4,37].

Our findings show whole chromosome gains in the MIN-Os and tumors to be fairly common, while no whole chromosome loss was observed. This is consistent with LOH analysis that did not find increased chromosome loss or large deletions in Tg(PyVmT) mammary tumor [38]. A previous CGH study by Hodgson et al. found that chromosomes 2, 11 and 15 most often had recurrent gains in PyVmT mammary tumors [24]. Indeed, the tumor samples we analyzed frequently had whole chromosome gains of these chromosomes, as well as chromosomes 1 and 10. However, there were no genetic changes found consistently during the transition from MIN-O to tumor in the different lines. Moreover, as expected from the CGH analysis, there seem to be no common expression changes that are associated with every MIN-O to tumor transition event.

The whole genome analysis with aCGH also led to the identification of areas of small deletions. Moreover, using a custom-designed array, the aberrations were confirmed and the breakpoints were refined. The smallest area confirmed by the zoom-in array was the 59,704 bp deletion on chromosome 3, which spans only one known gene, 2810410M20Rik. The zoom-in array clearly demonstrated the loss of 56 probes that represented this stretch of chromosome 3 in MIN-Os and the corresponding tumor samples. Moreover, for the other two areas of deletion, the losses of DNA were confirmed to span the same areas in the MIN-Os and the tumors. In general, the same small aberrations as well as large gains were seen in both MIN-Os and tumors, suggesting that these genomic changes were already present in the MIN-Os.

Chromosome aberrations in human breast cancer has been studied with LOH analysis [39] and array CGH [2,3,5,40-43]. These studies have identified regions of frequent copy number changes in breast cancer. For example, ErbB2 amplicon on 17q21 is amplified in 20–30% of breast cancers [44]. Frequent copy number changes of certain chromosome regions are associated with BRCA1 and BRCA2 tumors, and they can be classified by the genomic copy number alterations [40]. More recently, Fridlyand et al. has shown that breast tumors can be classified into three subtypes based on the copy number alteration phenotypes [45]. The "mixed amplifier" group had low level gains and losses with some recurring copy number changes, while the others two types had either few copy number changes ("1q/16q" group) or significant changes encompassing nearly 60% of the genome with copy number alterations ("complex" group). The patterns of genome alteration in our MIN-O lines resemble those of the first two groups. Interestingly, the majority of human tumors in these two groups were ER positive and that these two groups had better outcome compared to the complex group [45]. Our MIN-O lines may better represent human breast cancers that are low grade and less aggressive than those that are ER negative, high grade lesions with poor outcome.

### Major expression changes associated with tumorigenesis

Although there were no common genomic alterations found among all MIN-O samples examined, there seem to be common gene expression changes associated with the transition from normal mammary gland to MIN-O. For example, MIN-Os over-expressed cyclin D1 and Cdkn1a (P21). Cyclin D1 is a well-known oncogene, which is commonly over-expressed in human breast cancer [46]. Moreover, recent reports demonstrated that cyclin D1-associated kinase activity, through its association with CDK4, is required for ErbB2-dependent mammary tumorigenesis [47-49]. Cdkn1a (P21) was also significantly over-expressed in the MIN-Os. Cdkn1a is a cyclin-dependent kinase inhibitor and is commonly known to induce G1 arrest via the p53 pathway [50]. However this is not incongruous with Cdkn1a over-expression in the MIN-Os, as p21 is often over-expressed in human cancer, and more studies have shown p21 as a modulator of cell proliferation, rather than inhibitor [51,52]. Dysregulation of these genes in the MIN-Os demonstrates that although these tissues are phenotypically premalignant, the MIN-Os already have molecular changes known to be associated with tumorigenesis.

Since major molecular changes had occurred in the MIN-Os, tumors developed from the MIN-Os had surprisingly small genetic differences from the corresponding MIN-O tissues. Many genes coding for extracellular proteins were down-regulated in tumors. Among these genes, Lum and Gsn are two genes whose association with breast cancer has been well studied. Decreased expression of Lum is associated with poor outcome of invasive breast cancer [53] and down-regulation of Gsn is associated with breast cancer progression [54].

### Changes associated with aneuploidy

Each MIN-O line, established from a distinct MIN lesion in the original transgenic mammary fat pad, has distinct genomic and expression profiles and biological characteristics. This is also similar to human DCIS lesions, because DCIS is subclassified into different histological grades and the pattern of chromosome alterations and gene expression signatures differs between different histological grades [3,4]. MIN-Os can be classified into subgroups by genomic and molecular profiles that reflect biological behavior (metastatic potential, tumor latency, estrogen sensitivity).

Genetically unstable MIN-O lines, which have multiple whole chromosome gains, almost always had gain of chromosome 2. AurkA was one of the genes on chromosome 2 that was highly over-expressed in these MIN-Os. AurkA is localized to the spindles and its overexpression induces aberrant mitosis that results in centrosome duplication and aneuploidy [55]. Overexpression of AurkA is associated with cancer, including human DCIS [56,57]. Other genes involved in mitotic spindle assembly and centrosome duplication were also over-expressed in these MIN-Os. Ran, Kif22/Kid, Plk1 and Nde1 [58] are important for mitotic spindle assembly [58-61]. Mitotic spindle components, tublins alpha1 and beta6 were both over-expressed. Plk1 also regulates centrosome assembly and separation, and controls the onset of G2/M transition in the cell cycle by activating cdc25c [62]. Interestingly, beta-tubulin and cdc25c are substrates of Plk1 [61]. Other substrates of Plk1 are subunits of anaphase promoting complex (APC), which regulates progression and exit through mitosis by proteolysis of cell cycle regulators [59]. Two APC related genes, a component of APC, Apc11, and an activator of APC, cdc20, were both up-regulated in these MIN-Os. APC/cdc20 complex is important for metaphase to anaphase transition [59]. These genes highlight dysregulation of G2/M phase, especially during metaphase/anaphase when sister chromatids and spindle poles separate.

### Changes associated with metastasis

Highly metastatic MIN-O lines had alteration of genes involved in immune response and apoptotic processes. Recently, studies have shown that the inflammation process is closely linked to cancer development [63,64]. Innate immune systems promote tumorigenesis by producing factors that activate tumor associated cells and changes stromal microenvironment [65]. It is believed that changes in apoptotic tumor cell death pattern affect the tumor microenvironment and the associated immune response [66]. In particular, for the metastatic processes, tumor cells utilize many of the same factors, such as adhesion molecules, chemokines and receptors, used by inflammatory cells [67]. In the Tg(PyVmT) model, lack of functional macrophages inhibits the development of invasive mammary carcinoma and pulmonary metastasis [68], and other factors that regulate tumor microenvironment also affect the metastatic potential [69-72]. Gas6 is the ligand for Axl receptor tyrosine kinase, which regulates immune system and inhibits apoptosis [73,74]. Bcl6 is a transcriptional regulator that has an anti-apoptotic role and also affects humoral immune response [75]. Both of these genes were highly over-expressed in both metastatic MIN-O lines. Moreover, the expression of ICAM-1, an endothelial intercellular adhesion molecule was down-regulated in these MIN-Os. ICAM-1 is known to be down-regulated by tumor derived angiogenic factors in order to inhibit leukocyte infiltration and to escape from anti-tumor immune surveillance [76,77]. These expression changes suggest the alteration of the genes related to the immune modulation in these MIN-Os, which may contribute to the development of tumor associated environment that promote migration and invasion.

The "Metastatic signature gene set" [35] was not differentially expressed between the metastatic and non-metastatic MIN-O lines. This seems to be contradictory to the results by Qiu et al [36] in which the signature gene set was predictive of metastatic behaviour of Tg(PyVmT) tumors. However, this study compares MIN-Os with different metastatic potentials, which express PyVmT as the initiating oncogene in the same genetic background (FVB), and is fundamentally different from the study by Qiu et al in which Tg(PyVmT) tumors from various genetic backgrounds were compared. Thus, while the metastatic signature gene set may be effective in classifying metastatic disease from a group of genetically polymorphic samples, the MIN-O mouse lines will be useful for investigating specific molecular mechanisms responsible for metastasis in the disease tissues.

### Changes associated with estrogen dependence

ER status of the MIN-Os, in general, reflected the ovarian hormone sensitivity of the lesions. The 8w-B and 8w-D lines, which were associated with nuclear ER-positive MIN-O cells, were the most sensitive to the ovariectomy. In all four MIN-O lines studied, significantly less ER-positive cells were found in the tumors from the intact hosts, and in the MIN-Os from the ovariectomized hosts.

Two common functions that were affected in the estrogen sensitive MIN-O lines were steroid biosynthesis and angiogenesis. Significance of these two functions as well as the expression differences in the ERs and aromatase may represent the difference in estrogen mediated signaling in the 8w-B and D MIN-Os from the rest of the MIN-O lines, since estrogen modulates endothelial cell behavior, including angiogensis [78]. This concept is further supported by the dysregulation of genes that are known to be regulated by estrogen or ER, such as Cyp1b1 [79], Nf-kB2 [80], RelB, Strn [81], Fbln1 [82] or genes that interact with estrogen or ER, such as Abcg1, Gnai2, and Jun [83]. The down-regulation of two Nf-kB family members, Nf-kB2 and RelB, maybe significant since ER alpha is known to inhibit the Nf-kB activity by various mechanisms, including direct binding to the Nf-kB complex to inhibit transcriptional activation [80]. Jun was up-regulated in estrogen sensitive MIN-Os. ER activates transcription at AP-1 sites via interaction with Jun/Fos/coactivator complex [83]. Dysregulation of these genes seems to draw a picture of increase ER and estrogen activities in these MIN-Os.

## Conclusion

In summary, in this mouse model of breast cancer, the majority of the molecular changes responsible for tumorigenesis are already present in early premalignant lesions. The progression to invasive carcinoma is associated with only few additional changes, and these changes are not common in all tumors. Moreover, the molecular heterogeneity of the premalignant lesions represents phenotypic differences of these lesions. Thus, the MIN-O mouse model recapitulates both the preencoding and heterogeneity found in early human breast lesions and may be used as a high fidelity model for the biology of human breast cancer.

## List of abbreviations used

comparative genomic hybridization (CGH), ductal carcinoma in situ (DCIS), estrogen receptor (ER), invasive ductal carcinoma (IDC), mammary intraepithelial neoplasia outgrowth (MIN-O), MIN (mammary intraepithelial neoplasia), polyomavirus middle-T (PyVmT), array comparative genomic hybridization (aCGH), Gene Ontology (GO)

## Competing interests

The author(s) declare that they have no competing interests.

## Authors' contributions

RN participated in the ovariectomy study, performed RNA isolation and gene expression analysis, and drafted the manuscript. JM established the MIN-O lines, and designed and performed the ovariectomy experiment. RD performed the gene expression microarrays and the array CGH, and identified the chromosome aberrations. CB performed the GSR analysis and assisted with the gene expression analysis. SL was involved in transplant surgeries, MIN-O tissue collection and gene expression studies, as well as critical editing of this manuscript. CC performed the analysis of CGH data and designed the custom zoom-in arrays. LY established the MIN-O lines and performed MIN-O transplant surgeries. AB and RC assisted in the histology analysis. RC also directed the establishment of the MIN-O lines and made this study possible. JG directed this study and helped draft the manuscript. All authors read and approved the final manuscript.

## Pre-publication history

The pre-publication history for this paper can be accessed here:



## Supplementary Material

Additional File 1 **Cell cycle and cell death genes that are differentially expressed in MIN-Os**. Lists of cell cycle and cell death genes that were differentially expressed (p < 0.01) in the MIN-Os when compared to the prelactating normal mammary gland controls. Click here for file

Additional File 2 **Lists of tumors vs MIN-Os expression difference from each MIN-O line**. Expression profiles from tumors and MIN-Os from each MIN-O line were compared and significant differences were identified (p < 0.01). Click here for file

Additional File 3 **Differentially expressed genes on chromosome 2 that are common to MIN-O lines with whole chromosome losses**. Among the differentially expressed gene lists (p < 0.01) of 8w-B and 4w-4 MIN-O lines, genes on chromosome 2 that are common to both lists were identified. Click here for file

Additional File 4 **Differentially expressed genes (p < 0.01) that are specific to the two MIN-O lines with high metastatic index**. From the lists of differentially expressed genes from each MIN-O lines, genes that are only specific to 4w-11 and 8w-D were identified. Click here for file

Additional File 5 **Expression differences of ER and aromatase between ovarian hormone sensitive and less sensitive MIN-Os**. ERalpha, ERbeta, and aromatase expression differences were compared between estrogen sensitive (8w-B&8w-D) and less sensitive (4w-4&4w-11) MIN-O lines. Click here for file

Additional File 6 **Differentially expressed genes (p < 0.01) that are specific to estrogen sensitive MIN-Os**. From the lists of differentially expressed genes from each MIN-O lines, genes that are only specific to 8w-B and 8w-D were identified. Click here for file

## Figures and Tables

**Table 1:** Regions selected for high resolution custom CGH microarrays

**Chromosomal Interval**	**Base Pairs**	**RM Length**	**Cytobands**	**Total Probes**	**Genes**	**Probe Density (-RM)**
Chr2:130057657-134054840	3,997,184	1,593,942	qF1-qF2	4,393	97	547
Chr3:82669962-85356557	2,686,596	900,443	qE3-qF1	3,263	24	547
Chr11:42126777-46489372	4,362,596	1,560,848	qA5-qB1.1	5,120	49	547
Chr11:101602336-121653288	20,050,953	6,694,702	qD-qE2	24,413	584	547
Chr17:71771954-72520788	748,835	344,281	qE2	740	7	547

**Totals**	**31,846,164**	**11,094,216**		**37,929**	**671**	

Chr7:1-141,766,352	141,766,352	58,864,541		2,900		28,587
Chr8:1-127,874,053	127,874,053	48,159,999		2,090		38,141
Print Controls				1,370		

**Totals**				**44,289**		

**Table 2:** Characteristics of MIN-O lines

MIN-O line	Major histological characteristics of MIN-Os*	Metastasis Index*	ER status of MIN-Os
4w-4	Solid chords & nests	0	Negative or cytoplasmic
4w-11	Microacini & organized cysts	0.63	Mostly cytoplasmic
8w-B	Dysplastic cysts. Better differentiated. Microacinar structures.	0	Nuclear (+++)
8w-D	Prominent and distinct microacinar patterns. Organized cysts. Patches of eosinophilic microacini.	0.47	Nuclear (+)

* From Maglione et al 2004.

**Table 3:** Effect of ovariectomy on MIN-O growth and tumor latency

MIN-O line	MIN-O size at 5-weeks posttransplantation				Tumor latency (in weeks)		
				
		Ovx					
						
	Control %FPF (n)	% FPF (n)	relative to control	p-value	Control TE_50_	Ovx TE_50_	p-value
4w-4	55.00 ± 10.00 (4)	24.29 ± 9.76 (7)	0.44	*0.0008*	10.1	>10.1	0.113
4w-11	37.50 ± 17.08 (4)	15.00 ± 18.17 (6)	0.40	0.08	10.2	12.1	0.118
8w-B*	67.50 ± 12.58 (4)	22.86 ± 7.56 (7)	0.34	*<0.0001*	4.9	11.1	*0.002*
8w-D	60.00 ± 0.00 (4)	10.71 ± 4.50 (7)	0.18	*<0.0001*	8.15	12.1	*0.017*

* from Namba et al 2005

**Table 4:** Tumor vs MIN-O expression differences

**Probe Set ID**	**Gene Title**	**Gene Symbol**	**FC in tumors**	**GO biological function**
99104_at	adiponectin, C1Q and collagen domain containing	Adipoq	-21.75	Lipid metabolism, Organic acid metabolism
99671_at	adipsin	Adn	-35.98	
98924_at	ADP-ribosyltransferase 3	Art3	-2.55	
92537_g_at	adrenergic receptor, beta 3	Adrb3	-2.88	
102327_at	amine oxidase, copper containing 3	Aoc3	-3.66	Cell adhesion
96785_at	ankyrin repeat domain 47	Ankrd47	-2.04	
104761_at	anthrax toxin receptor 2	Antxr2	-1.60	
160375_at	carbonic anhydrase 3	Car3	-20.83	
101539_f_at	carboxylesterase 3	Ces3	-4.06	Lipid metabolism, Organic acid metabolism
102016_at	cell death-inducing DFFA-like effector c	Cidec	-4.79	
95415_f_at	complement component 1, r subcomponent	C1r	-2.64	
92224_at	C-type lectin domain family 3, member b	Clec3b	-5.87	
96346_at	cysteine dioxygenase 1, cytosolic	Cdo1	-9.76	Organic acid metabolism
93996_at	cytochrome P450, family 2, subfamily e, polypeptide 1	Cyp2e1	-3.54	
96742_at	dermatopontin	Dpt	-5.06	Cell adhesion
103031_g_at	dynamin 1	Dnm1	-2.21	
92535_at	early B-cell factor 1	Ebf1	-1.81	
100567_at	fatty acid binding protein 4, adipocyte	Fabp4	-2.31	
92441_at	fibroblast activation protein	Fap	-2.27	
101991_at	flavin containing monooxygenase 1	Fmo1	-3.20	
93040_at	FXYD domain-containing ion transport regulator 1	Fxyd1	-2.69	
93750_at	gelsolin	Gsn	-5.43	
102373_at	glutamyl aminopeptidase	Enpep	-2.86	
99108_s_at	growth hormone receptor	Ghr	-1.99	
92833_at	histidine ammonia lyase	Hal	-2.25	Organic acid metabolism
95546_g_at	insulin-like growth factor 1	Igf1	-2.07	
103904_at	insulin-like growth factor binding protein 6	Igfbp6	-4.34	
92366_at	laminin, alpha 2	Lama2	-3.25	Cell adhesion
103083_at	lipase, hormone sensitive	Lipe	-2.46	Lipid metabolism
160083_at/95611_at	lipoprotein lipase	Lpl	-5.18	Lipid metabolism
93353_at	lumican	Lum	-9.33	
97520_s_at	neuronatin	Nnat	-5.92	
100120_at	nidogen 1	Nid1	-2.19	Cell adhesion
160481_at	phosphoenolpyruvate carboxykinase 1, cytosolic	Pck1	-2.61	Lipid metabolism, Organic acid metabolism
102763_at	plasma membrane associated protein, S3-12	MGI:1929709	-8.13	
93826_at	protein phosphatase 2, regulatory subunit B (B56), alpha isoform	Ppp2r5a	-1.43	
102366_at	resistin	Retn	-10.89	
97835_at	retinoic acid receptor responder (tazarotene induced) 2	Rarres2	-6.19	
96047_at	retinol binding protein 4, plasma	Rbp4	-4.08	
104445_at	RIKEN cDNA 4631408O11 gene	4631408O11Rik	-5.20	
101861_at	sarcoglycan, epsilon	Sgce	-1.62	
102707_f_at	serine (or cysteine) peptidase inhibitor, clade A, member 3C	Serpina3c	-1.94	
97429_at	SNF related kinase	Snrk	-1.65	
92582_at	solute carrier family 1 (neutral amino acid transporter), member 5	Slc1a5	-1.94	
160320_at	sorbin and SH3 domain containing 1	Sorbs1	-2.74	Cell adhesion
94056_at/94057_g_at	stearoyl-Coenzyme A desaturase 1	Scd1	-2.76	Lipid metabolism, Organic acid metabolism
103087_at	sulfotransferase family 1A, phenol-preferring, member 1	Sult1a1	-1.68	Lipid metabolism
103568_at	sushi-repeat-containing protein	Srpx	-2.84	
104280_at	synuclein, gamma	Sncg	-4.63	
102916_s_at/161907_s_at	tenascin XB	Tnxb	-4.73	Cell adhesion
160547_s_at	thioredoxin interacting protein	Txnip	-2.83	
99052_at	zinc finger homeobox 1a	Zfhx1a	-1.63	

**Table 5:** Chromosome 2 gene expression in MIN-O lines with whole chromosome gains (8w-B & 4w-4 vs. 8w-D & 4w-11)

		# of probes		
	p-value	increased	decreased	total probes
Chr 2	<0.01	60	5	871
	<0.05	143	15	

**Table 6:** Chromosome 1, 10 and 11 gene expression differences in MIN-O lines with whole chromosome gains (8w-B vs. 8w-D, 4w-4 & 4w-11)

		# of probes		
	p-value	increased	decreased	total probes
Chr 1	<0.01	78	11	652
	<0.05	217	24	
Chr 10	<0.01	61	4	481
	<0.05	139	14	
Chr 11	<0.01	121	11	957
	<0.05	255	36	

**Table 7:** Expression in the deleted region (2F1-2, chr2: 130,621,986-131,735,939)

Genes	U74Av2 probe ID	lower expression in 8w-D MIN-Os*
Hspa12b		
1700037H04Rik	97210_at	x
Spef1/4931426K16Rik	93475_at	x
Cenpb	99486_at	x
cdc25b	99012_at	
2310035K24Rik	96819_at	
D430028G21Rik		
Pank2		
Rnf24	97200_f_at	x
Smox		
Adra1d		
1600014E20Rik	161097_at	
Prnp	100606_at	x
Prnd		
Rassf2	94771_at	
Slc23a2	104267_at	x

* determined by 8w-D MIN-Os compared to rest of the MIN-O samples

**Table 8:** Expression in the duplicated region (11B, ch11:42,631,278-44,744,905)

Genes	U74Av2 probe ID	higher expression in 5189MINO**
Pttg1	101026_at	x
D11Ertd730e	160772_I_at	x
	161428_at	x
C1qtnf2		
Fabp6	99977_at	x
Ttc1	96921_at	x
	161887_r_at	x
Adra1b	92340_at	x
Il12b	100779_at	x
Ublcp1	94865_at	x
3732413l11Rik	96104_at	x
Ebf1	92535_at	x

** determined by 5189 MIN-O compared to 5207 MIN-O

**Table 9:** Significant biological functions for MIN-O vs PL expression differences for 4w-4 line

**Name**	**GO ID**	**Corrected P-value**
**Highly significant GO functions**		
DNA-dependent DNA replication	GO:0006261	0.00E+00
organic acid biosynthesis	GO:0016053	0.00E+00
chromatin assembly or disassembly	GO:0006333	0.00E+00
nucleosome assembly	GO:0006334	0.00E+00
regulation of cyclin dependent protein kinase activity	GO:0000079	2.68E-02

**Table 10:** Significant biological functions for MIN-O vs PL expression differences for 4w-11 line

**Name**	**GO ID**	**Corrected P-value**
**I-kappB/NF-kappaB**		
regulation of I-kappaB kinase/NF-kappaB cascade	GO:0043122	0.00E+00
positive regulation of I-kappaB kinase/NF-kappaB cascade	GO:0043123	0.00E+00
I-kappaB kinase/NF-kappaB cascade	GO:0007249	0.00E+00
**apoptotic program**		
apoptotic program	GO:0008632	0.00E+00
**Immune response**		
lymphocyte differentiation	GO:0030098	7.04E-03
antigen presentation, exogenous antigen	GO:0019884	1.59E-02
xenobiotic metabolism	GO:0006805	2.02E-02
T-cell activation	GO:0042110	2.72E-02
response to xenobiotic stimulus	GO:0009410	2.68E-02
T-cell differentiation	GO:0030217	2.71E-02
response to bacteria	GO:0009617	2.87E-02
regulation of immune response	GO:0050776	3.35E-02
defense response to bacteria	GO:0042742	3.87E-02
antigen presentation	GO:0019882	4.21E-02
**Reponse to radiation/UV**		
response to radiation	GO:0009314	6.37E-03
phototransduction	GO:0007602	1.53E-02
detection of light	GO:0009583	1.91E-02
response to light	GO:0009416	2.79E-02

**Table 11:** Significant biological functions for MIN-O vs PL expression differences for 8w-B line

**Name**	**GO ID**	**Corrected P-value**
**Spindle/Microtubule**		
mitotic spindle organization and biogenesis	GO:0007052	0.00E+00
spindle organization and biogenesis	GO:0007051	0.00E+00
microtubule cytoskeleton organization and biogenesis	GO:0000226	0.00E+00
microtubule-based movement	GO:0007018	4.70E-07
chromosome segregation	GO:0007059	4.32E-03
microtubule polymerization	GO:0046785	1.59E-02
microtubule polymerization or depolymerization	GO:0031109	1.97E-02
M phase specific microtubule process	GO:0000072	3.32E-02
**Cell cycle**		
regulation of mitosis	GO:0007088	0.00E+00
cell cycle checkpoint	GO:0000075	0.00E+00
interphase of mitotic cell cycle	GO:0051329	0.00E+00
G1/S transition of mitotic cell cycle	GO:0000082	0.00E+00
negative regulation of cell cycle	GO:0045786	6.08E-03
G2/M transition of mitotic cell cycle	GO:0000086	1.29E-02
mitotic checkpoint	GO:0007093	1.28E-02
regulation of cyclin dependent protein kinase activity	GO:0000079	1.25E-02
G1 phase of mitotic cell cycle	GO:0000080	1.41E-02
traversing start control point of mitotic cell cycle	GO:0007089	1.67E-02
cell cycle arrest	GO:0007050	1.67E-02
mitotic anaphase	GO:0000090	2.63E-02
anaphase	GO:0051322	2.72E-02
prophase	GO:0051324	2.81E-02
**Angiogenesis**		
blood vessel morphogenesis	GO:0048514	6.37E-03
angiogenesis	GO:0001525	1.01E-02
blood vessel development	GO:0001568	9.73E-03
**Steroid**		
steroid biosynthesis	GO:0006694	3.45E-02

**Table 12:** Significant biological functions for MIN-O vs PL expression differences for 8w-D line

**Name**	**GO ID**	**Corrected P-value**
**Response to radiation/UV**		
phototransduction	GO:0007602	0.00E+00
detection of light	GO:0009583	0.00E+00
response to radiation	GO:0009314	0.00E+00
response to light	GO:0009416	0.00E+00
response to UV	GO:0009411	2.88E-02
**Apoptosis**		
apoptotic program	GO:0008632	0.00E+00
**Immune response**		
complement activation, classical pathway	GO:0006958	9.56E-03
positive regulation of immune response	GO:0050778	1.15E-02
regulation of immune response	GO:0050776	1.36E-02
T-cell activation	GO:0042110	2.23E-02
antigen processing	GO:0030333	2.25E-02
cellular defense response (sensu Vertebrata)	GO:0016066	2.32E-02
antimicrobial humoral response	GO:0019730	2.58E-02
positive regulation of lymphocyte activation	GO:0051251	2.60E-02
xenobiotic metabolism	GO:0006805	2.58E-02
symbiotic interaction between host and other organism	GO:0044404	2.56E-02
oxygen and reactive oxygen species metabolism	GO:0006800	3.21E-02
complement activation	GO:0006956	3.21E-02
response to xenobiotic stimulus	GO:0009410	3.97E-02
lymphocyte differentiation	GO:0030098	3.94E-02
pathogenesis	GO:0009405	3.96E-02
T-helper 1 type immune response	GO:0042088	4.19E-02
detection of biotic stimulus	GO:0009595	4.49E-02
cytokine and chemokine mediated signaling pathway	GO:0019221	4.72E-02
antigen presentation, endogenous antigen	GO:0019883	4.71E-02
lymphocyte proliferation	GO:0046651	4.90E-02
antigen presentation	GO:0019882	8.19E-03
**Angiogenesis**		
blood vessel morphogenesis	GO:0048514	8.92E-03
blood vessel development	GO:0001568	1.17E-02
angiogenesis	GO:0001525	1.47E-02
**ECM**		
extracellular structure organization and biogenesis	GO:0043062	3.18E-02
extracellular matrix organization and biogenesis	GO:0030198	3.15E-02
cell-matrix adhesion	GO:0007160	4.31E-02
**Signaling pathways**		
I-kappaB kinase/NF-kappaB cascade	GO:0007249	0.00E+00
regulation of I-kappaB kinase/NF-kappaB cascade	GO:0043122	2.38E-02
positive regulation of I-kappaB kinase/NF-kappaB cascade	GO:0043123	2.35E-02
Wnt receptor signaling pathway	GO:0016055	1.16E-04
Ras protein signal transduction	GO:0007265	4.61E-03
inactivation of MAPK	GO:0000188	2.43E-02
**Steroid**		
steroid biosynthesis	GO:0006694	3.27E-06
sterol metabolism	GO:0016125	4.16E-02

## References

[B1] Fearon ER, Vogelstein B (1990). A genetic model for colorectal tumorigenesis. Cell.

[B2] Chin K, de Solorzano CO, Knowles D, Jones A, Chou W, Rodriguez EG, Kuo WL, Ljung BM, Chew K, Myambo K, Miranda M, Krig S, Garbe J, Stampfer M, Yaswen P, Gray JW, Lockett SJ (2004). In situ analyses of genome instability in breast cancer. Nat Genet.

[B3] Hwang ES, DeVries S, Chew KL, Moore DH, Kerlikowske K, Thor A, Ljung BM, Waldman FM (2004). Patterns of chromosomal alterations in breast ductal carcinoma in situ. Clin Cancer Res.

[B4] Ma XJ, Salunga R, Tuggle JT, Gaudet J, Enright E, McQuary P, Payette T, Pistone M, Stecker K, Zhang BM, Zhou YX, Varnholt H, Smith B, Gadd M, Chatfield E, Kessler J, Baer TM, Erlander MG, Sgroi DC (2003). Gene expression profiles of human breast cancer progression. Proc Natl Acad Sci U S A.

[B5] Aubele M, Mattis A, Zitzelsberger H, Walch A, Kremer M, Welzl G, Hofler H, Werner M (2000). Extensive ductal carcinoma In situ with small foci of invasive ductal carcinoma: evidence of genetic resemblance by CGH. Int J Cancer.

[B6] Perou CM, Sorlie T, Eisen MB, van de Rijn M, Jeffrey SS, Rees CA, Pollack JR, Ross DT, Johnsen H, Akslen LA, Fluge O, Pergamenschikov A, Williams C, Zhu SX, Lonning PE, Borresen-Dale AL, Brown PO, Botstein D (2000). Molecular portraits of human breast tumours. Nature.

[B7] Sorlie T, Perou CM, Tibshirani R, Aas T, Geisler S, Johnsen H, Hastie T, Eisen MB, van de Rijn M, Jeffrey SS, Thorsen T, Quist H, Matese JC, Brown PO, Botstein D, Eystein Lonning P, Borresen-Dale AL (2001). Gene expression patterns of breast carcinomas distinguish tumor subclasses with clinical implications. Proc Natl Acad Sci U S A.

[B8] van 't Veer LJ, Dai H, van de Vijver MJ, He YD, Hart AA, Mao M, Peterse HL, van der Kooy K, Marton MJ, Witteveen AT, Schreiber GJ, Kerkhoven RM, Roberts C, Linsley PS, Bernards R, Friend SH (2002). Gene expression profiling predicts clinical outcome of breast cancer. Nature.

[B9] Mallon E, Osin P, Nasiri N, Blain I, Howard B, Gusterson B (2000). The basic pathology of human breast cancer. J Mammary Gland Biol Neoplasia.

[B10] Shoker BS, Sloane JP (1999). DCIS grading schemes and clinical implications. Histopathology.

[B11] Maglione JE, McGoldrick ET, Young LJ, Namba R, Gregg JP, Liu L, Moghanaki D, Ellies LG, Borowsky AD, Cardiff RD, MacLeod CL (2004). Polyomavirus middle T-induced mammary intraepithelial neoplasia outgrowths: single origin, divergent evolution, and multiple outcomes. Mol Cancer Ther.

[B12] Namba R, Maglione JE, Young LJ, Borowsky AD, Cardiff RD, MacLeod CL, Gregg JP (2004). Molecular characterization of the transition to malignancy in a genetically engineered mouse-based model of ductal carcinoma in situ. Mol Cancer Res.

[B13] Cardiff RD, Anver MR, Gusterson BA, Hennighausen L, Jensen RA, Merino MJ, Rehm S, Russo J, Tavassoli FA, Wakefield LM, Ward JM, Green JE (2000). The mammary pathology of genetically engineered mice: the consensus report and recommendations from the Annapolis meeting. Oncogene.

[B14] Maglione JE, Moghanaki D, Young LJ, Manner CK, Ellies LG, Joseph SO, Nicholson B, Cardiff RD, MacLeod CL (2001). Transgenic Polyoma middle-T mice model premalignant mammary disease. Cancer Res.

[B15] Namba R, Young LJ, Maglione JE, McGoldrick ET, Liu S, Wurz GT, DeGregorio MW, Borowsky AD, MacLeod CL, Cardiff RD, Gregg JP (2005). Selective estrogen receptor modulators inhibit growth and progression of premalignant lesions in a mouse model of DCIS. Breast Cancer Res.

[B16] RepeatMasker. http://www.repeatmasker.org.

[B17] Hughes TR, Mao M, Jones AR, Burchard J, Marton MJ, Shannon KW, Lefkowitz SM, Ziman M, Schelter JM, Meyer MR, Kobayashi S, Davis C, Dai H, He YD, Stephaniants SB, Cavet G, Walker WL, West A, Coffey E, Shoemaker DD, Stoughton R, Blanchard AP, Friend SH, Linsley PS (2001). Expression profiling using microarrays fabricated by an ink-jet oligonucleotide synthesizer. Nat Biotechnol.

[B18] Agilent Technologies. http://www.chem.agilent.com.

[B19] Barrett MT, Scheffer A, Ben-Dor A, Sampas N, Lipson D, Kincaid R, Tsang P, Curry B, Baird K, Meltzer PS, Yakhini Z, Bruhn L, Laderman S (2004). Comparative genomic hybridization using oligonucleotide microarrays and total genomic DNA. Proc Natl Acad Sci U S A.

[B20] ErmineJ software. http://microarray.cu-genome.org/ermineJ/.

[B21] Lee HK, Braynen W, Keshav K, Pavlidis P (2005). ErmineJ: tool for functional analysis of gene expressiono data sets. BMC Bioinformatics.

[B22] UCSC Genome Browser. http://genome.ucsc.edu/cgi-bin/hgGateway.

[B23] Lin EY, Jones JG, Li P, Zhu L, Whitney KD, Muller WJ, Pollard JW (2003). Progression to malignancy in the polyoma middle T oncoprotein mouse breast cancer model provides a reliable model for human diseases. Am J Pathol.

[B24] Hodgson JG, Malek T, Bornstein S, Hariono S, Ginzinger DG, Muller WJ, Gray JW (2005). Copy number aberrations in mouse breast tumors reveal loci and genes important in tumorigenic receptor tyrosine kinase signaling. Cancer Res.

[B25] Anderson DM, Maraskovsky E, Billingsley WL, Dougall WC, Tometsko ME, Roux ER, Teepe MC, DuBose RF, Cosman D, Galibert L (1997). A homologue of the TNF receptor and its ligand enhance T-cell growth and dendritic-cell function. Nature.

[B26] Lim KH, Baines AT, Fiordalisi JJ, Shipitsin M, Feig LA, Cox AD, Der CJ, Counter CM (2005). Activation of RalA is critical for Ras-induced tumorigenesis of human cells. Cancer Cell.

[B27] Gery S, Tanosaki S, Bose S, Bose N, Vadgama J, Koeffler HP (2005). Down-regulation and growth inhibitory role of C/EBPalpha in breast cancer. Clin Cancer Res.

[B28] Grimm SL, Rosen JM (2003). The role of C/EBPbeta in mammary gland development and breast cancer. J Mammary Gland Biol Neoplasia.

[B29] Shi B, Vinyals A, Alia P, Broceno C, Chen F, Adrover M, Gelpi C, Price JE, Fabra A (2006). Differential expression of MHC class II molecules in highly metastatic breast cancer cells is mediated by the regulation of the CIITA transcription Implication of CIITA in tumor and metastasis development. Int J Biochem Cell Biol.

[B30] Tamiolakisl D, Venizelos I, Lambropoulou M, Jivannakis T, Seliniotakis E, Tsikouras P, Limberis V, Tsalkidis A, Papadopoulos N (2004). Gains and losses of HLA class II (DR) and CD4 in atypical hyperplasia, carcinoma in situ and infiltrating ductal carcinoma of the breast. Acta Medica (Hradec Kralove).

[B31] Eckerdt F, Yuan J, Strebhardt K (2005). Polo-like kinases and oncogenesis. Oncogene.

[B32] Kinch MS, Carles-Kinch K (2003). Overexpression and functional alterations of the EphA2 tyrosine kinase in cancer. Clin Exp Metastasis.

[B33] Ren B, Cam H, Takahashi Y, Volkert T, Terragni J, Young RA, Dynlacht BD (2002). E2F integrates cell cycle progression with DNA repair, replication, and G(2)/M checkpoints. Genes Dev.

[B34] Holland SJ, Powell MJ, Franci C, Chan EW, Friera AM, Atchison RE, McLaughlin J, Swift SE, Pali ES, Yam G, Wong S, Lasaga J, Shen MR, Yu S, Xu W, Hitoshi Y, Bogenberger J, Nor JE, Payan DG, Lorens JB (2005). Multiple roles for the receptor tyrosine kinase axl in tumor formation. Cancer Res.

[B35] Ramaswamy S, Ross KN, Lander ES, Golub TR (2003). A molecular signature of metastasis in primary solid tumors. Nat Genet.

[B36] Qiu TH, Chandramouli GV, Hunter KW, Alkharouf NW, Green JE, Liu ET (2004). Global expression profiling identifies signatures of tumor virulence in MMTV-PyMT-transgenic mice: correlation to human disease. Cancer Res.

[B37] Schuetz CS, Bonin M, Clare SE, Nieselt K, Sotlar K, Walter M, Fehm T, Solomayer E, Riess O, Wallwiener D, Kurek R, Neubauer HJ (2006). Progression-specific genes identified by expression profiling of matched ductal carcinomas in situ and invasive breast tumors, combining laser capture microdissection and oligonucleotide microarray analysis. Cancer Res.

[B38] Ritland SR, Rowse GJ, Chang Y, Gendler SJ (1997). Loss of heterozygosity analysis in primary mammary tumors and lung metastases of MMTV-MTAg and MMTV-neu transgenic mice. Cancer Res.

[B39] Wang ZC, Lin M, Wei LJ, Li C, Miron A, Lodeiro G, Harris L, Ramaswamy S, Tanenbaum DM, Meyerson M, Iglehart JD, Richardson A (2004). Loss of heterozygosity and its correlation with expression profiles in subclasses of invasive breast cancers. Cancer Res.

[B40] Jonsson G, Naylor TL, Vallon-Christersson J, Staaf J, Huang J, Ward MR, Greshock JD, Luts L, Olsson H, Rahman N, Stratton M, Ringner M, Borg A, Weber BL (2005). Distinct genomic profiles in hereditary breast tumors identified by array-based comparative genomic hybridization. Cancer Res.

[B41] Gunther K, Merkelbach-Bruse S, Amo-Takyi BK, Handt S, Schroder W, Tietze L (2001). Differences in genetic alterations between primary lobular and ductal breast cancers detected by comparative genomic hybridization. J Pathol.

[B42] Yao J, Weremowicz S, Feng B, Gentleman RC, Marks JR, Gelman R, Brennan C, Polyak K (2006). Combined cDNA array comparative genomic hybridization and serial analysis of gene expression analysis of breast tumor progression. Cancer Res.

[B43] O'Connell P (2003). Genetic and cytogenetic analyses of breast cancer yield different perspectives of a complex disease. Breast Cancer Res Treat.

[B44] Slamon DJ, Godolphin W, Jones LA, Holt JA, Wong SG, Keith DE, Levin WJ, Stuart SG, Udove J, Ullrich A (1989). Studies of the HER-2/neu proto-oncogene in human breast and ovarian cancer. Science.

[B45] Fridlyand J, Snijders AM, Ylstra B, Li H, Olshen A, Segraves R, Dairkee S, Tokuyasu T, Ljung BM, Jain AN, McLennan J, Ziegler J, Chin K, Devries S, Feiler H, Gray JW, Waldman F, Pinkel D, Albertson DG (2006). Breast tumor copy number aberration phenotypes and genomic instability. BMC Cancer.

[B46] Sutherland RL, Musgrove EA (2002). Cyclin D1 and mammary carcinoma: new insights from transgenic mouse models. Breast Cancer Res.

[B47] Landis MW, Pawlyk BS, Li T, Sicinski P, Hinds PW (2006). Cyclin D1-dependent kinase activity in murine development and mammary tumorigenesis. Cancer Cell.

[B48] Reddy HK, Mettus RV, Rane SG, Grana X, Litvin J, Reddy EP (2005). Cyclin-dependent kinase 4 expression is essential for neu-induced breast tumorigenesis. Cancer Res.

[B49] Yu Q, Sicinska E, Geng Y, Ahnstrom M, Zagozdzon A, Kong Y, Gardner H, Kiyokawa H, Harris LN, Stal O, Sicinski P (2006). Requirement for CDK4 kinase function in breast cancer. Cancer Cell.

[B50] Liu G, Lozano G (2005). p21 stability: linking chaperones to a cell cycle checkpoint. Cancer Cell.

[B51] Weiss RH (2003). p21Waf1/Cip1 as a therapeutic target in breast and other cancers. Cancer Cell.

[B52] Yuste L, Montero JC, Esparis-Ogando A, Pandiella A (2005). Activation of ErbB2 by overexpression or by transmembrane neuregulin results in differential signaling and sensitivity to herceptin. Cancer Res.

[B53] Troup S, Njue C, Kliewer EV, Parisien M, Roskelley C, Chakravarti S, Roughley PJ, Murphy LC, Watson PH (2003). Reduced expression of the small leucine-rich proteoglycans, lumican, and decorin is associated with poor outcome in node-negative invasive breast cancer. Clin Cancer Res.

[B54] Winston JS, Asch HL, Zhang PJ, Edge SB, Hyland A, Asch BB (2001). Downregulation of gelsolin correlates with the progression to breast carcinoma. Breast Cancer Res Treat.

[B55] Meraldi P, Honda R, Nigg EA (2002). Aurora-A overexpression reveals tetraploidization as a major route to centrosome amplification in p53-/- cells. Embo J.

[B56] Hoque A, Carter J, Xia W, Hung MC, Sahin AA, Sen S, Lippman SM (2003). Loss of aurora A/STK15/BTAK overexpression correlates with transition of in situ to invasive ductal carcinoma of the breast. Cancer Epidemiol Biomarkers Prev.

[B57] Miyoshi Y, Iwao K, Egawa C, Noguchi S (2001). Association of centrosomal kinase STK15/BTAK mRNA expression with chromosomal instability in human breast cancers. Int J Cancer.

[B58] Feng Y, Walsh CA (2004). Mitotic spindle regulation by Nde1 controls cerebral cortical size. Neuron.

[B59] Castro A, Bernis C, Vigneron S, Labbe JC, Lorca T (2005). The anaphase-promoting complex: a key factor in the regulation of cell cycle. Oncogene.

[B60] Peters JM (2002). The anaphase-promoting complex: proteolysis in mitosis and beyond. Mol Cell.

[B61] van Vugt MA, Medema RH (2005). Getting in and out of mitosis with Polo-like kinase-1. Oncogene.

[B62] Myer DL, Bahassi el M, Stambrook PJ (2005). The Plk3-Cdc25 circuit. Oncogene.

[B63] Coussens LM, Werb Z (2002). Inflammation and cancer. Nature.

[B64] Philip M, Rowley DA, Schreiber H (2004). Inflammation as a tumor promoter in cancer induction. Semin Cancer Biol.

[B65] Bissell MJ, Radisky D (2001). Putting tumours in context. Nat Rev Cancer.

[B66] Zeh HJ, Lotze MT (2005). Addicted to death: invasive cancer and the immune response to unscheduled cell death. J Immunother.

[B67] Muller A, Homey B, Soto H, Ge N, Catron D, Buchanan ME, McClanahan T, Murphy E, Yuan W, Wagner SN, Barrera JL, Mohar A, Verastegui E, Zlotnik A (2001). Involvement of chemokine receptors in breast cancer metastasis. Nature.

[B68] Lin EY, Pollard JW (2004). Macrophages: modulators of breast cancer progression. Novartis Found Symp.

[B69] Forrester E, Chytil A, Bierie B, Aakre M, Gorska AE, Sharif-Afshar AR, Muller WJ, Moses HL (2005). Effect of conditional knockout of the type II TGF-beta receptor gene in mammary epithelia on mammary gland development and polyomavirus middle T antigen induced tumor formation and metastasis. Cancer Res.

[B70] Muraoka-Cook RS, Kurokawa H, Koh Y, Forbes JT, Roebuck LR, Barcellos-Hoff MH, Moody SE, Chodosh LA, Arteaga CL (2004). Conditional overexpression of active transforming growth factor beta1 in vivo accelerates metastases of transgenic mammary tumors. Cancer Res.

[B71] Muraoka RS, Dumont N, Ritter CA, Dugger TC, Brantley DM, Chen J, Easterly E, Roebuck LR, Ryan S, Gotwals PJ, Koteliansky V, Arteaga CL (2002). Blockade of TGF-beta inhibits mammary tumor cell viability, migration, and metastases. J Clin Invest.

[B72] Bugge TH, Lund LR, Kombrinck KK, Nielsen BS, Holmback K, Drew AF, Flick MJ, Witte DP, Dano K, Degen JL (1998). Reduced metastasis of Polyoma virus middle T antigen-induced mammary cancer in plasminogen-deficient mice. Oncogene.

[B73] Lee WP, Wen Y, Varnum B, Hung MC (2002). Akt is required for Axl-Gas6 signaling to protect cells from E1A-mediated apoptosis. Oncogene.

[B74] Schwartzberg PL (2001). Immunology. Tampering with the immune system. Science.

[B75] Phan RT, Dalla-Favera R (2004). The BCL6 proto-oncogene suppresses p53 expression in germinal-centre B cells. Nature.

[B76] Bouma-ter Steege JC, Baeten CI, Thijssen VL, Satijn SA, Verhoeven IC, Hillen HF, Wagstaff J, Griffioen AW (2004). Angiogenic profile of breast carcinoma determines leukocyte infiltration. Clin Cancer Res.

[B77] Griffioen AW, Damen CA, Martinotti S, Blijham GH, Groenewegen G (1996). Endothelial intercellular adhesion molecule-1 expression is suppressed in human malignancies: the role of angiogenic factors. Cancer Res.

[B78] Cid MC, Schnaper HW, Kleinman HK (2002). Estrogens and the vascular endothelium. Ann N Y Acad Sci.

[B79] Tsuchiya Y, Nakajima M, Yokoi T (2005). Cytochrome P450-mediated metabolism of estrogens and its regulation in human. Cancer Lett.

[B80] Kalaitzidis D, Gilmore TD (2005). Transcription factor cross-talk: the estrogen receptor and NF-kappaB. Trends Endocrinol Metab.

[B81] Lu Q, Pallas DC, Surks HK, Baur WE, Mendelsohn ME, Karas RH (2004). Striatin assembles a membrane signaling complex necessary for rapid, nongenomic activation of endothelial NO synthase by estrogen receptor alpha. Proc Natl Acad Sci U S A.

[B82] Moll F, Katsaros D, Lazennec G, Hellio N, Roger P, Giacalone PL, Chalbos D, Maudelonde T, Rochefort H, Pujol P (2002). Estrogen induction and overexpression of fibulin-1C mRNA in ovarian cancer cells. Oncogene.

[B83] Kushner PJ, Agard DA, Greene GL, Scanlan TS, Shiau AK, Uht RM, Webb P (2000). Estrogen receptor pathways to AP-1. J Steroid Biochem Mol Biol.

